# In Vivo Antidiabetic and Antilipidemic Effect of Thiazolidine-2,4-Dione Linked Heterocyclic Scaffolds in Obesity-Induced Zebrafish Model

**DOI:** 10.3390/ph18071023

**Published:** 2025-07-10

**Authors:** Asmaa Galal-Khallaf, Dawlat Mousa, Aml Atyah, Mohamed El-Bahnsawye, Mona K. Abo Hussein, Ibrahim El Tantawy El Sayed, Elshaymaa I. Elmongy, Reem Binsuwaidan, Abdel Moneim A. K. El-Torgoman, Hamed Abdel-Bary, Khaled Mohammed-Geba

**Affiliations:** 1 Zoology Department, Faculty of Science, Menoufia University, Shebin EL-Kom P.O. Box 32511, Egypt; as_kh_22@yahoo.com (A.G.-K.); khaledmohammed@science.menofia.edu.eg (K.M.-G.); 2 Chemistry Department, Faculty of Science, Menoufia University, Shebin El-Kom P.O. Box 32511, Egypt; dawlatmousa92@gmail.com (D.M.); aml7amed731997@gmail.com (A.A.); m_nh22010@yahoo.com (M.E.-B.); ibrahimtantawy@yahoo.co.uk (I.E.T.E.S.); torgoman43@yahoo.com (A.M.A.K.E.-T.); hamedabdelbary2006@yahoo.com (H.A.-B.); 3 Clinical Microbiology and Immunology Department, National Liver Institute, Menoufia University, Shebin El-Kom P.O. Box 32511, Egypt; monaabohussein@yahoo.com; 4 Department of Pharmaceutical Chemistry, Faculty of Pharmacy, Helwan University, Ain Helwan, Cairo P.O. Box 11795, Egypt; 5 Department of Pharmaceutical Sciences, College of Pharmacy, Princess Nourah bint Abdulrahman University, P.O. Box 84428, Riyadh 11671, Saudi Arabia; rabinsuwaidan@pnu.edu.sa

**Keywords:** acridine, antihyperglycemic, gene expression, hybrids, hyperlipidemic, neocryptolepine, thiazolidinedione, zebrafish

## Abstract

**Background:** Type 2 diabetes mellitus (T2DM) presents a significant global health challenge, with obesity being a major contributing risk factor alongside genetic and non-genetic elements. Current treatments focus on reducing hyperglycemia and preventing T2DM progression, often involving drug combinations for enhanced efficacy. This study introduces two novel nitrogen-containing heterocyclic scaffolds: neocryptolepine–thiazolidinedione (NC-TZD) **8** and acridine–thiazolidinedione (AC-TZD) **11**. **Methods:** These compounds were synthesized and characterized using various spectroscopic techniques. Their antihyperglycemic and antihyperlipidemic effects were assessed in an obesity-induced zebrafish model. Hyperglycemia was induced by immersing zebrafish in 100 mM glucose monohydrate for two weeks. Fish were then divided into groups receiving either 20 mg or 80 mg of the drugs per kg of body weight, alongside negative and positive control groups. **Results:** Both doses of hybrids **8** and **11** effectively restored glucose, triglyceride, insulin, and nuclear factor kappa beta (*nfκβ*) mRNA levels to normal. However, only the lower doses restored peroxisomal acyl-CoA oxidase (*acox1*) mRNA levels, with higher doses proving less effective. A molecular modeling study supported the antidiabetic potential of hybrids **8** and **11**, suggesting interactions with target proteins PPAR-α and *acox1*. In silico ADMET analysis revealed promising oral bioavailability and drug likeness for both compounds. **Conclusions:** The findings indicate that both hybrids exhibit significant antihyperglycemic and antihypertriglyceridemic effects, particularly at lower doses. These results highlight the promising therapeutic potential of these novel oral bioavailable compounds in managing T2DM. Further research is warranted to elucidate their mechanisms of action.

## 1. Introduction

Diabetes mellitus (DM) is a chronic metabolic disorder associated with life-threatening risk factors as mentioned by the WHO in 2024. In 2021, approximately 537 million people aged between 20 and 79 were diabetics, with a predicted rise in this number to 643 million by 2030 and 783 million by 2045. This is expected to create an escalating challenge for both patients and healthcare providers.

There are 73 million diabetics in the Middle East and North Africa, which makes this region the third most impacted in the world with T2DM, following the Western Pacific and Southeast Asia [1]. Diabetes induces significant economic losses in all countries, especially middle-income and developing countries. This is because of their greater population densities, which support a quick, poorly controlled rise in its prevalence in comparison to that in developed countries [2]. This raises concerns about a larger burden in the future of societal and economic consequences. While access to health facilities and the purchase of common antidiabetic drugs consume a large portion of family savings in middle-income and developing countries, global health costs associated with diabetes are largely driven by more health visits, diabetes-related complications, lengthy hospital stays, paramedical care, and uptake of newer, more expensive diagnostic and therapeutic tools in high-income country settings [2].

There are two main types of DM with different etiologies, type 1 (T1DM) and type 2 (T2DM). T1DM is a long-term autoimmune illness in which insulin-producing pancreatic cells are selectively destroyed. T1DM occurs because of an autoimmune disease, in which the body’s immune system gradually destroys the pancreatic beta islets of Langerhans, reducing insulin production. Both environmental and genetic predisposing factors have a role in the development of T1DM, but the exact relationships of those are not yet understood [3]. Meanwhile, despite T2DM also exhibiting genetic and environmental backgrounds, it seems that they differ completely from those leading to T1DM. For example, the β islets of Langerhans do not express MHC class II [4]. T2DM onset occurs upon the failure of pancreatic β cells to produce enough insulin to meet the demands of insulin-responsive tissues, which safely store and metabolize glucose. In comparison to T1DM, many genomic loci could be related to β cells’ dysfunction in T2DM, in addition to epigenomic factors like chromatin hypomethylation [5]. Many studies agree that obesity and an unfavorable lifestyle (e.g., smoking, alcohol consumption, irregular physical activity, and imbalanced diet) could be related to a high risk of incidence of T2DM, sometimes regardless of a genetic predisposition [6].

**Figure 1:**
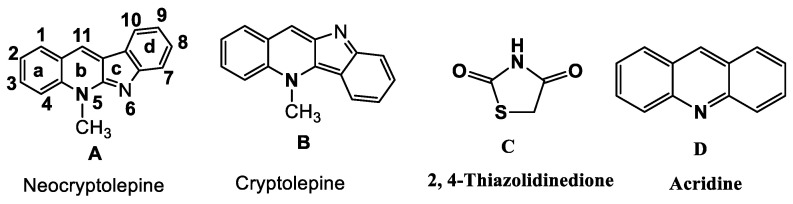
Selected nitrogen-containing heterocycle templates with potential biological activity.

In T2DM, insulin resistance precedes hyperglycemia, i.e., extremely elevated blood glucose levels over the normal reference range [7]. Fasting hyperglycemia appears due to elevated hepatic glucose production because of the relatively low insulin levels combined with hepatic insulin resistance [7]. Hyperglycemia is the main target for T2DM therapies for several reasons. For example, it has negative impacts on the immune system, which render T2DM patients more susceptible to infections [8]. Also, it can be related to a reduction in muscular mass, i.e., sarcopenia [9], as well as cardiovascular disorders and neurological and nephrological malfunctioning [10]. Moreover, hyperglycemia is a key factor in diabetic neuropathy’s development, a direct cause of excessive production of reactive oxygen metabolites, and an inhibitor of antioxidative mechanisms through non-enzymatic glycosylation of antioxidant enzymes [11]. Neocryptolepine (5-methyl-5*H*-indolo[2,3-b]quinoline) **A** (Figure 1) is a natural product alkaloid, isolated from the aqueous extracts of the West African climbing shrub *Cryptolepis sanguinolenta,* and has received notable attention in the literature as a consequence of its numerous biological activities. This makes neocryptolepine core an ideal starting point for the development of novel drug candidates [12,13,14]. In this context, several analogues have been synthesized based on this biologically active indoloquinoline alkaloid, and most of them exhibited a wide range of biological activities as anticancer, antiparasitic, anti-inflammatory, antibacterial, and topoisomerase inhibitors [12]. The antihyperglycemic and antihyperlipidemic activities of neocryptolepine have not been reported until now. However, the antihyperglycemic activity of the regioisomer cryptolepine **B** (Figure 1)-based analogues has already been reported [15].

In nature, many pharmacologically active compounds are produced, containing thiazolidinedione scaffold **C**. Molecules containing these core structure have been designed and synthesized, and they show a broad range of biological activity in the treatment of various clinical disorders [16]. In addition, thiazolidinedione TZD moiety **C** served as a powerful agent in the treatment of diabetes [17]. It is also known as glitazones and is a collection of a five-membered thiazolidinedione TZD moiety **C** with a couple of carbonyl groups at positions 2 and 4 [18]. It has shown antihyperglycemic and antilipidemic activity [19]. On the other hand, acridine core structure **D** and its analogues are one of the most successful classes of chemotherapeutic agents. It is generally acknowledged that acridine-based chemotherapeutic agents possess a broad capability in terms of influencing physiological and pathological processes, with wide medical applications including antidiabetic potential [20]. In this context, in drug design and discovery, molecular hybridization has emerged as a potent tool that presents an attractive strategy for obtaining more effective drugs to treat a wide range of human diseases, including diabetes mellitus [21]. One technique to develop hybrid molecules is to utilize a linker chain to integrate two or more drug pharmacophores into a single multifunctional molecule. The primary objectives of this pharmacophore merging approach are to decrease drug–drug interactions, increase biological activity and specificity, decrease known side effects associated with each hybrid part, and interact with two or more targets [22]. Given the information above, one could infer that incorporating biologically active N-heterocyclic compounds of natural origin, like neocryptolepine and acridine, into the thiazolidinedione core structure may enhance both antidiabetic and antihyperlipidemic effects, resulting in a synergistic impact compared to using each component individually. The present study focuses on the preliminary in vivo screening of novel TZD-linked N-heterocyclic scaffolds in a diabetic zebrafish model. The zebrafish model is increasingly validated in the literature as a reliable, cost-effective, and ethically favorable alternative to mammalian models for metabolic disorder studies [23]. Our choice of this model allows for rapid screening of antidiabetic activity of newly synthesized hybrids. The hybridization of the two pharmacophoric moieties, neocryptolepine and acridine, may potentiate antidiabetic activity. In this study, we will combine these two scaffolds into a single molecular framework to explore their potential synergistic effects. The rational selection of target hybrids is based on their well-known pharmacological profile and predicted interactions with a biological target. This approach aims to optimize the chances of finding a molecule that effectively interacts with the target, leading to the desired therapeutic effects. In addition, a molecular docking study was executed to confirm the binding of conjugates with the target protein structures.

## 2. Results

### 2.1. Synthesis and Elucidation of the New Compounds

**Scheme 1:**
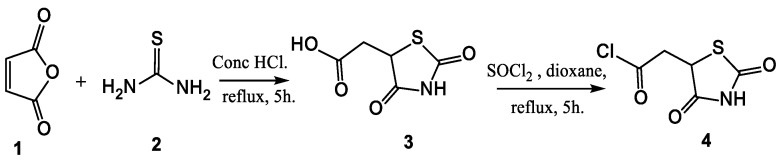
Synthesis of the 2-(2,4-dioxothiazolidin-5-yl)acetyl chloride **4**.

The synthesis of 2, 4-thiazolidinedione-5-acetic acid **3** was achieved with a good yield through the reaction between maleic anhydride **1** and thiourea **2**, utilizing concentrated hydrochloric acid. Additionally, when compound **3** was treated with SOCl_2_ in dry 1,4-dioxane, it resulted in the formation of acid chloride **4** with a high yield [24], Scheme 1.

**Scheme 2:**
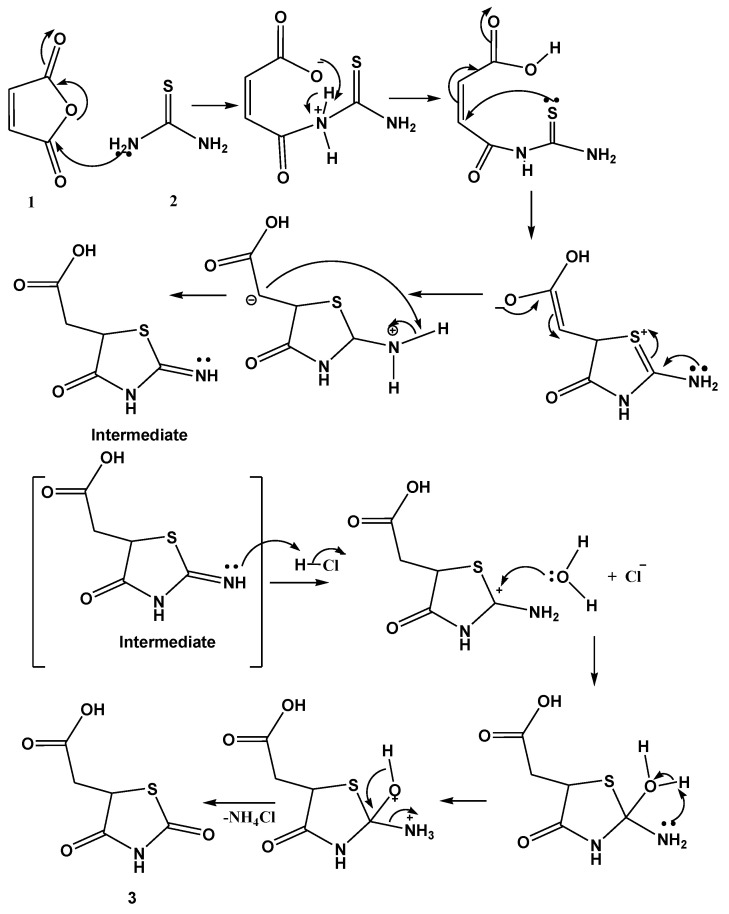
Plausible mechanism for the synthesis of the intermediate 2,4-thiazolidinedione-5-acetic acid **3**.

To form the starting 2,4-thiazolidinedione-5-acetic acid **3**, the proposed mechanism includes two crucial steps. In the first step, the cyclic anhydride is opened by nucleophilic thiourea nitrogen followed by the addition of nucleophilic sulfur to an α, β-unsaturated Michael acceptor through a Michael addition. Moreover, compound **3** is synthesized via intramolecular cyclization, and then it undergoes acid hydrolysis, as depicted in Scheme 2.

**Scheme 3:**
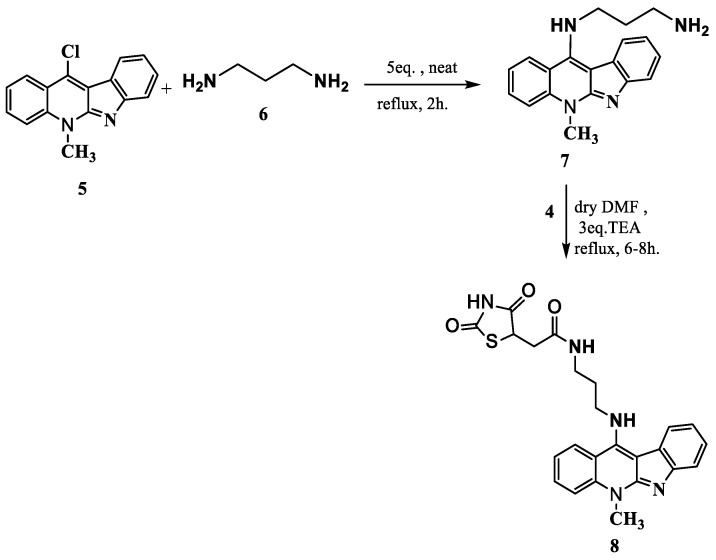
Synthesis of neocryptolepine–thiazolidinedione (NC-TZD) **8**.

**Scheme 4:**
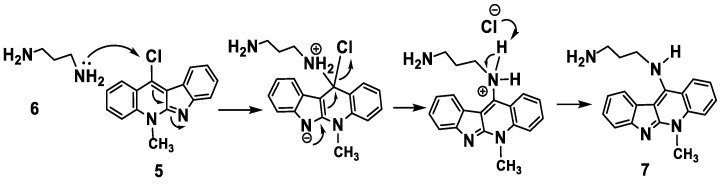
Proposed mechanism of aromatic nucleophilic substitution for formation of **7**.

The corresponding 11-aminopropylaminoneocryptolepine derivative **7** was obtained by condensing 11-chloroneocryptolepine **5** with five equivalents of 1,3-diaminopropane **6** in neat reaction under heating for 2 h, as shown in Scheme 3. The dimer formation has been avoided completely by using five equivalents of bisamine which provides best solvation condition for derivative **5**. The reaction was found to be finished and monitored by TLC in 2hr yielding 94%. This process is known as a nucleophilic aromatic substitution (SN_Ar_) reaction mechanism, in which the amino group of **6** is substituted with the chlorine atom at the unsaturated sp^2^ C-11 position and C-9 position, as shown in Scheme 4.

This reaction produces the end product **7** by first adding the amino group (: Nu–) to create a resonance-stabilized anion with a new C–N bond and then eliminating HCl as the triethyl amine hydrochloride salt. FT-IR and NMR were two of the spectroscopic techniques used to clarify the synthesized 11-amino alkyl amino neocryptolepine free amines **7**. The (NH) functional group’s absorption bands were visible at 3216 cm^−1^ in the FT-IR spectrum. Conversely, the ^1^H-NMR spectra revealed the characteristic (N-CH_3_) of the neocryptolepine core, which has been reported as 4.24 ppm for **7**. On the other hand, the (N-CH_3_) group in ^13^C-NMR was found at 41.56 ppm for **7**.

**Scheme 5:**
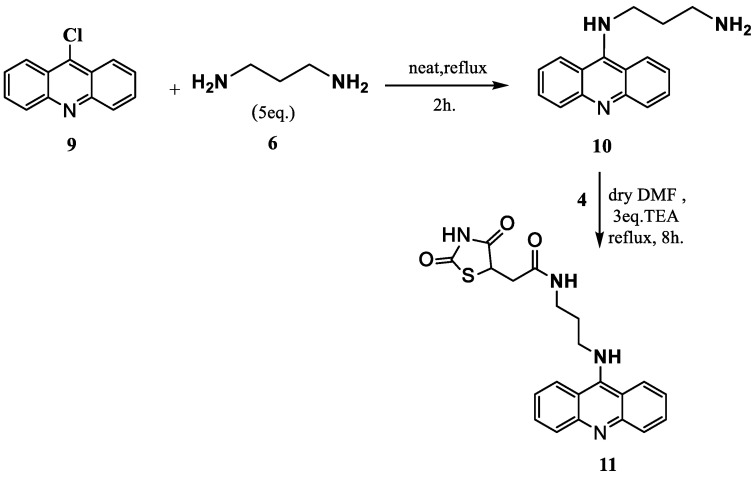
Synthesis of acridine–thiazolidinedione (AC-TZD) **11**.

Moreover, the requested N1-(acridin-9-yl) propane-1,3-diamine **10** is synthesized by reacting 9-chloroacridine **9** with 5 molar excess of 1,3-diaminopropane **6** in a neat reaction under a reflux condition to give monomer **10** in a good yield, as depicted in Scheme 5.

It was found that not only does increasing the amount of amine plays an important role in minimizing the dimer formation but also the reaction time is also crucial. Further optimization of the reaction conditions indicated that optimum results are obtained after running the amination reaction using 5 molar equivalents from the amine for 2 h.

It is worth noting that the reaction of 11-chloroneocryptolepine (**5**) and 9-chloroacridine (**9**) with 1,3-diaminopropane (**6**), as illustrated in Scheme 3 and Scheme 5, afforded the corresponding amino analogues **7** and **10** (the monomeric products). Although dimer formation was expected during the reaction, we successfully minimized this by optimizing the conditions: conducting the reaction under solvent-free (neat) conditions and using a five-fold excess of diamine **6**. Under these optimized conditions, the reaction was complete, with no evidence of dimer formation, as confirmed by thin-layer chromatography (TLC). The proposed mechanism of dimer formation highlights the role of two nucleophiles: the primary diamine, 1,3-diaminopropane (**6**), and the in situ-formed monomer (**7** or **10**). The latter, due to its steric bulk and reduced nucleophilicity, is less reactive. Its steric hindrance helps limit further reaction, thereby favoring monomer formation over dimerization.

**Scheme 6:**
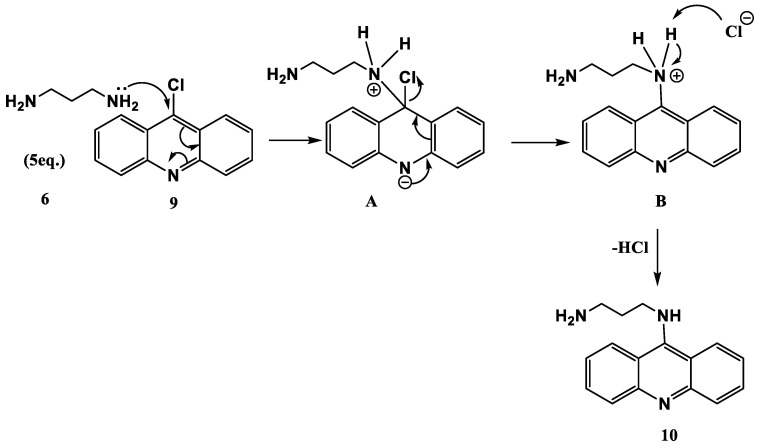
Proposed mechanism for the suggested mechanism for synthesis of **10**.

Spectroscopic analysis confirmed the structure of compound **10**. Infrared (IR) spectroscopy revealed absorption bands between 3232 and 3417 cm^−1^, indicating the presence of NH and NH_2_ groups. Proton nuclear magnetic resonance (^1^H-NMR) spectroscopy showed exchangeable peaks between 9.31 and 12.64 ppm, characteristic of NH_2_ and NH groups. Mass spectrometry detected the expected molecular ion peaks, confirming the formation of the target compound. The proposed mechanism for the formation of compound **10** involves a nucleophilic aromatic substitution (SNAr) reaction. Specifically, the amine nitrogen attacks the chloride ion at the C-9 position of the acridine core, forming a resonance-stabilized anion and a new C–N bond (as illustrated in Scheme 6).

#### Formation of Neocryptolepine–thiazolidinedione (NC-TZD) **8** and Acridine–thiazolidinedione (AC-TZD) **11**

Compounds **7** (N-(3-Aminopropyl)-5-methyl-5H-indolo[2,3-b]quinolin-11-amine) and **10** (N1-(acridin-9-yl)propane-1,3-diamine) were reacted with 2-(2,4-dioxothiazolidin-5-yl)acetyl chloride 4 in a 1:1 molar ratio. The reaction was performed in dry dimethylformamide (DMF) with a threefold excess of triethylamine (TEA) as a base, under reflux conditions. This yielded the new compounds **8** (2-(2,4-dioxothiazolidin-5-yl)-N-(3-((5-methyl-5H-indolo[2,3-b]quinolin-11-yl)amino)propyl)acetamide) and **11** (N-(acridin-9-yl)-2-(2,4-dioxothiazolidin-5-yl)acetamide), respectively. The structures of the newly synthesized compounds **8** and **11** were confirmed using IR, ^1^H NMR, ^13^C NMR, and mass spectroscopy. IR spectra showed strong absorption bands in the range of 3320–3565 cm^−1^, attributed to NH groups. Additionally, absorption peaks in the range of 1743–1772 cm^−1^ were assigned to amido C=O groups. ^1^H-NMR spectra exhibited exchangeable signals between 5.83 and 12.11 ppm, corresponding to NH groups, and a singlet at δ 4.68 ppm, indicating the presence of an active methylene proton. Furthermore, the mass spectral analysis displayed the expected molecular ion peaks, which confirmed the formation of the expected products.

### 2.2. In Vivo Effects of the Compound on Zebrafish

#### 2.2.1. Assessment of Glucose (a) and Triglycerides (b) in Zebrafish Livers

**Figure 2:**
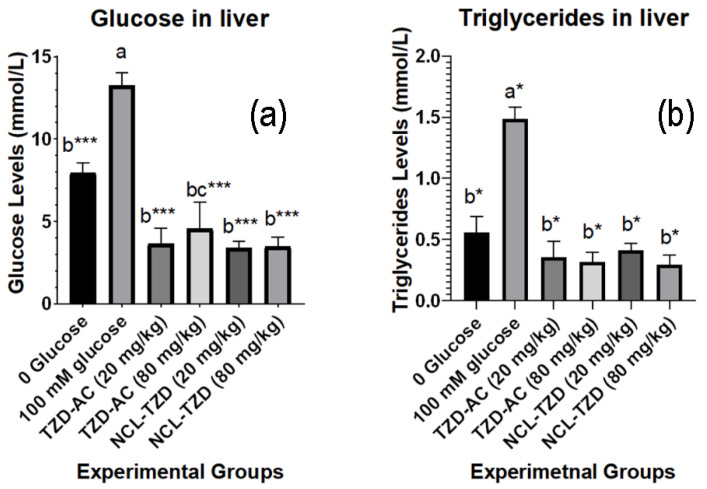
Levels (mmol L-1) of glucose (**a**) and triglycerides (**b**) in zebrafish livers in the experimental groups: 0 mM glucose (negative control), 100 mM glucose (positive control), 100 mM glucose-then-thiazolidine + acridine (TZD-Ac) oral administration (20 mg/kg and 80 mg/kg fish weights)m and 100 mM glucose-then-neocryptolepine + thiazolidine (NCL-TZD) oral administration (20 mg/kg and 80 mg/kg fish weights). Results are represented as mean ± standard error of means (SEM). Different black letters (a,b,c) above bars of each experimental group refer to significant differences between each other and relative to the positive control. One asterisk (*): *p* < 0.05. Triple asterisk (***): *p* < 0.01.

The positive control group for TZD-AC exhibited a significant elevation in comparison to all other groups (Figure 2a). Levels of glucose in the negative control group were about 5-fold lower than these of the positive control group. The low and high doses of both TZD-AC and NCL-TZD showed a significant reduction in glucose levels, to be at the same level as the negative control group (Figure 2a). Likewise, the positive control group showed a significant elevation of triglycerides, whereas both drugs at both doses reduced these levels significantly (Figure 2b).

#### 2.2.2. Assessment of Levels of Insulin in Zebrafish Liver Homogenates

**Figure 3:**
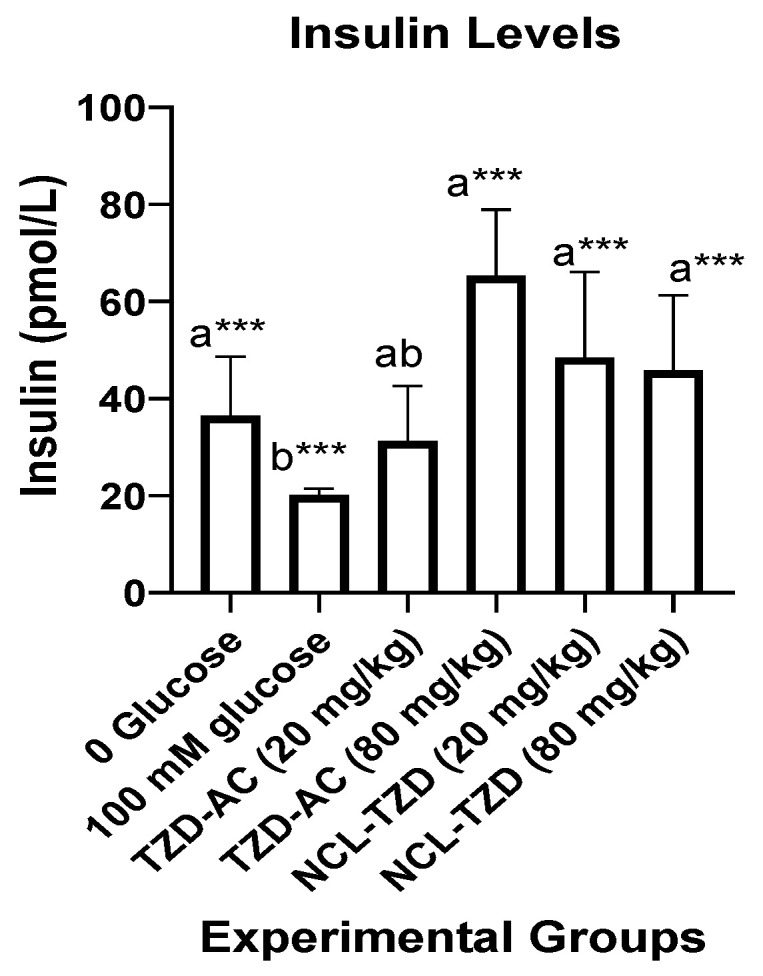
Levels (pmol L^−1^) of insulin in zebrafish liver homogenates in the following experimental groups: 0 mM glucose (negative control), 100 mM glucose (positive control), 100 mM glucose-then-thiazolidine + acridine (TZD-Ac) oral administration (20 mg/kg and 80 mg/kg fish weights), and 100 mM glucose-then-neocryptolepine + thiazolidine (NCL-TZD) oral administration (20 mg/kg and 80 mg/kg fish weights). Results are represented as mean ± standard error of means (SEM). Different black letters (a,b) above bars of each experimental group refer to significant differences between each other and relative to the positive control. Triple asterisk (***): *p* < 0.01.

Insulin levels dropped significantly in the group to which we kept administering glucose continuously, i.e., the positive control, from the negative control levels. However, both drugs could restore these insulin levels. Interestingly, insulin levels in the groups treated by the two drugs, at the two doses of each, exhibited higher insulin levels than the negative control. However, these levels were significantly high in response to the high doses of both drugs (Figure 3).

#### 2.2.3. Assessment of mRNA Levels (∆∆C_t_) of Nuclear Factor Kappa Beta (*nfκβ*) in Zebrafish Liver Homogenates

**Figure 4:**
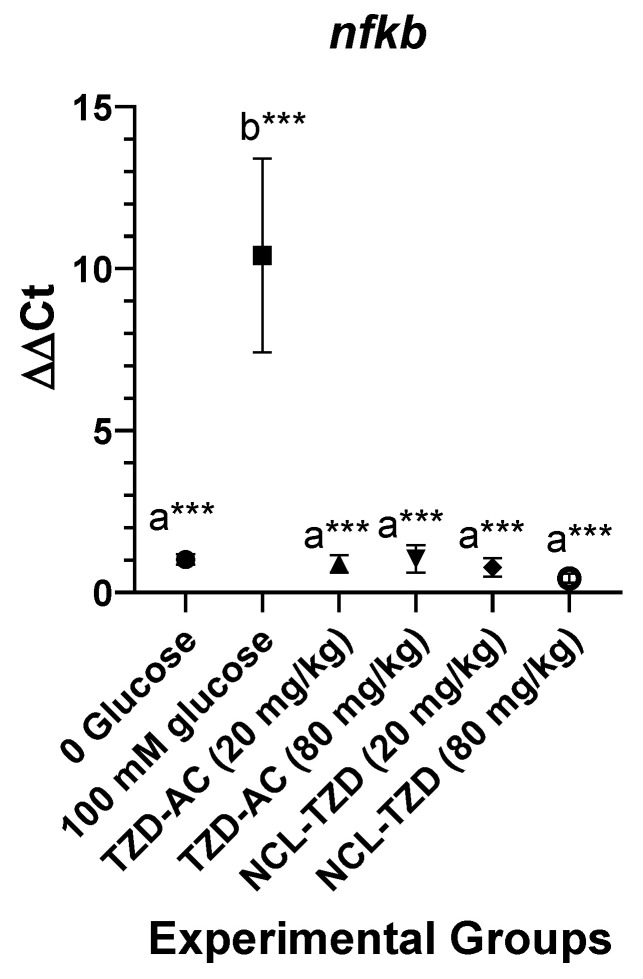
mRNA levels (∆∆C_t_) of nuclear factor kappa beta (*nfκβ*) in zebrafish liver homogenates in the following experimental groups: 0 mM glucose (negative control), 100 mM glucose (positive control), 100 mM glucose-then-thiazolidine + acridine (TZD-Ac) oral administration (20 mg/kg and 80 mg/kg fish weights), and 100 mM glucose-then-neocryptolepine + thiazolidine (NCL-TZD) oral administration (20 mg/kg and 80 mg/kg fish weights). Results are represented as mean ± standard error of means (SEM). Different black letters (a,b) above bars of each experimental group refer to significant differences between each other and relative to the positive control. Triple asterisk (***): *p* < 0.01.

mRNA levels of the main inflammatory mediator, i.e., *nfkb*, exhibited patterns that were very similar to those of glucose and triglycerides. The group kept continuously in glucose showed a significantly higher level than all other groups. Meanwhile, both TZD-AC at 20 mg kg^−1^ fish weight and 80 mg kg^−1^ fish weight and NCL-TZD at the same doses could restore the mRNA levels of *nfkb* to their normal control levels (Figure 4).

#### 2.2.4. Assessment of mRNA Levels (∆∆C_t_) of the Peroxisomal Acyl-Coenzyme A Oxidase 1 (*acox1*) in Zebrafish Liver Homogenates

**Figure 5:**
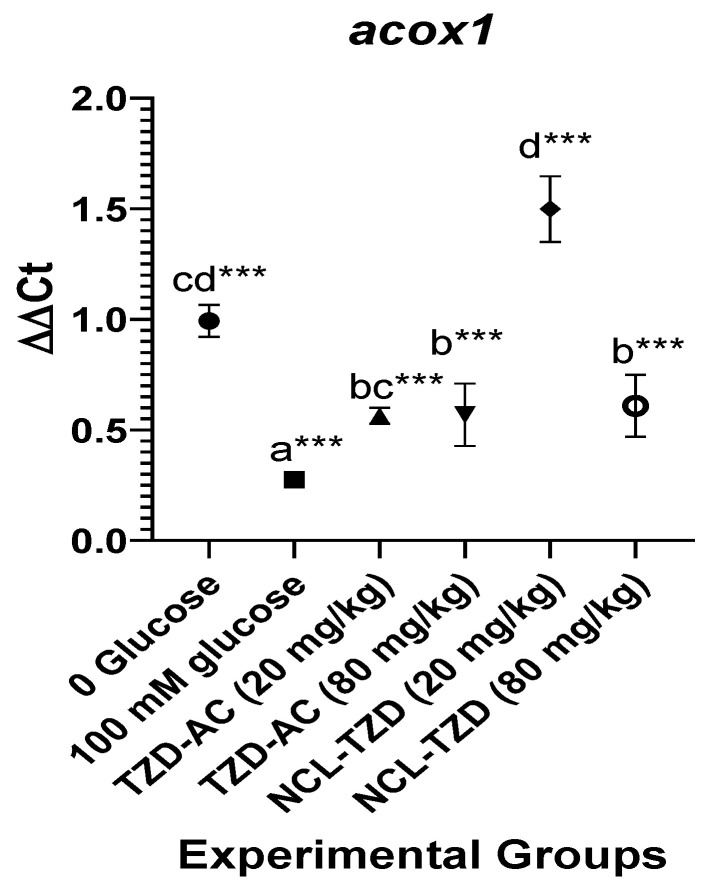
mRNA levels (∆∆C_t_) of the peroxisomal acyl-coenzyme A oxidase 1 (*acox1*) in zebrafish liver homogenates in the following experimental groups: 0 mM glucose (negative control), 100 mM glucose (positive control), 100 mM glucose-then-thiazolidine + acridine (TZD-Ac) oral administration (20 mg/kg and 80 mg/kg fish weights), and 100 mM glucose-then-neocryptolepine + thiazolidine (NCL-TZD) oral administration (20 mg/kg and 80 mg/kg fish weights). Results are represented as mean ± standard error of means (SEM). Different black letters (a,b,c,d) above bars of each experimental group refer to significant differences between each other and relative to the positive control. Triple asterisk (***): *p* < 0.01.

mRNA levels of *acox1* showed a more significant reduction in the positive control group than the negative control one. The low dose of TZD-AC drug showed a significant increase in *acox1*. However, these levels were kept significantly lower than the negative control group. Likewise, both the TZD-AC and NCL-TZD high-dose-treated groups showed significantly higher *acox1* levels than the positive group, yet they were still lower than the negative control group. The low dose of NCL-TZD, however, showed a several-fold increase in *acox1* expression levels, over both the positive and the negative control groups (Figure 5).

#### 2.2.5. Assessment of mRNA Levels (∆∆C_t_) of the Acetyl-CoA Carboxylase Alpha (*accα*) in Zebrafish Liver Homogenates

**Figure 6:**
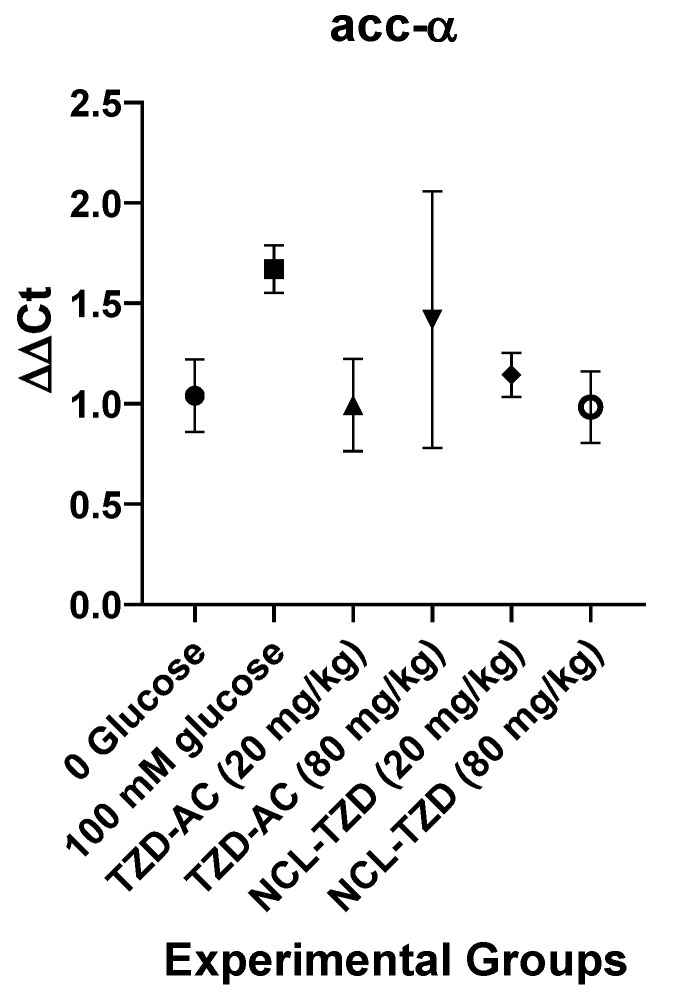
mRNA levels (∆∆C_t_) of the acetyl-CoA carboxylase alpha (*accα*) in zebrafish liver homogenates in the following experimental groups: 0 mM glucose (negative control), 100 mM glucose (positive control), 100 mM glucose then thiazolidine + acridine (TZD-Ac) oral administration (20 mg/kg and 80 mg/kg fish weights), and 100 mM glucose then neocryptolepine + thiazolidine (NCL-TZD) oral administration (20 mg/kg and 80 mg/kg fish weights). Results are represented as mean ± standard error of means (SEM). No significant differences among groups could be identified.

Finally, *accα* levels did not show significant differences (*p* = 0.6). However, some trends were found, with the positive control levels exceeding all other groups. The group treated with the high dose of the TZD-AC drug showed individually varying levels of *acc-α,* with some attaining the levels of the negative control group (no glucose) and others having *accα* levels equal or superior to the positive control group (i.e., glucose all the experiment) (Figure 6).

### 2.3. Docking Study

**Figure 7:**
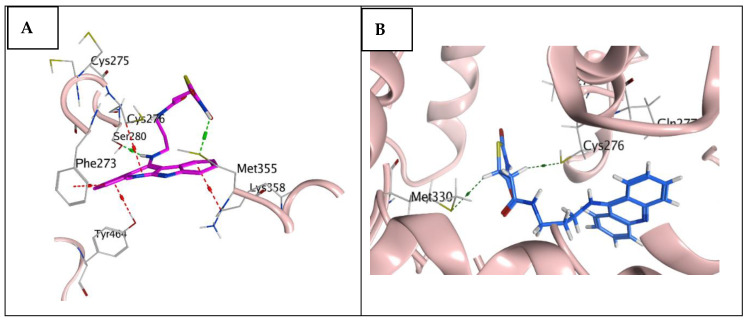
Docking on protein PDB ID 2ZNN (**A**): Compound **8** illustrated in pink sticks, and (**B**): compound **11** represented as blue sticks docked at the ligand binding site of the target protein PPAR-α represented as light pink ribbon, where H-bonds are in green dashed lines and hydrophobic interactions are in red dashed lines.

**Figure 8:**
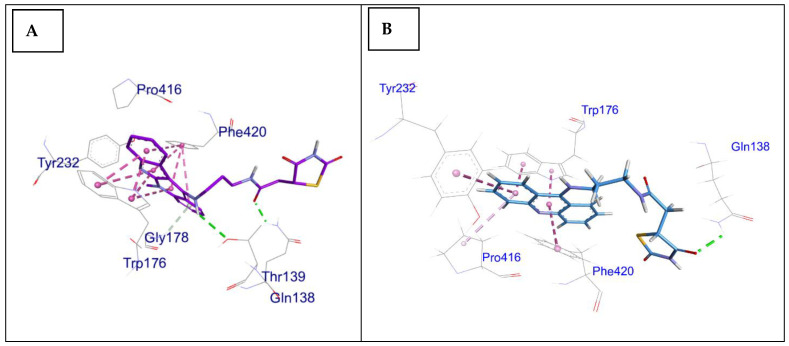
Docking on *ACOX1,* where (**A**) compound **8** is illustrated in magenta sticks docked at the ligand binding site, and (**B**) compound **11** is illustrated in blue sticks; all hydrogen bonds are represented in green dashed lines, and hydrophobic interactions are in pink dashed lines.

Compounds **8** and **11** were docked into the target protein for PPAR-α at the ligand binding cavity, showing binding energies of −8.59 and −7.89 kcal/mol, respectively, with a perfect fit not exceeding 1.8 Å (Table 1). The inhibition constant (Ki) of the prepared hybrids **8** and **11** was calculated, where compound **8** showed lower Ki values against the two target proteins than its analogue compound **11** (Table 1). Amino acid residues involved in interactions are alike those binding with the co-crystalized ligand of the downloaded protein, in particular, MET 330, SER 280 and TYR 464 in the binding pocket (Figure 7). However, upon docking of compounds **8** and **11** in the binding pocket of the second target ACOX1, the recorded binding energies were −8.51 and −7.15 kcal/mol, respectively, with an occupation of the ligand binding site with a root mean square deviation of less than 2 Å. Compound **8** showed two hydrogen bonds, with GLN 138 and THR 139, while compound **11** showed one hydrogen bond, with GLN 138 residue at the binding pocket, as shown in Figure 8.

### 2.4. In Silico ADMET Analysis for Compounds ***8*** and ***11***

In silico investigation of absorption, distribution, metabolism, and excretion (ADME) is important in early phases of drug development [25,26,27,28]. Key absorption parameters including intestinal absorption, skin sensitization, and oral bioavailability were analyzed (Table 2). Both compounds under study demonstrated intestinal absorption rates exceeding the reported standard 30%, achieving absorbance of 92% and 83% for compounds **8** and **11**, respectively.

The compounds tested showed skin permeability, with scores around −2.7 cm/h. Moreover, both compounds under study displayed a low human colon adenocarcinoma permeability (Caco-2) score of 0.6 for compound **8** and a slightly higher score of 1.0 for compound **11** when compared to the reference, which states that high permeability occurs when the Caco-2 value is greater than 0.9 [25,28].

Regarding the distribution of compounds, the volume of distribution (VDss), blood–brain barrier (BBB) permeability, and central nervous system (CNS) permeability were the parameters evaluated in silico. The VDss values indicated distribution volumes of 0.165 and 0.35 log L/kg for compounds **8** and **11**, respectively. Compound **8** had a log BB score of −0.987 while compound **11** recorded a score of −1.102. The log permeability surface (PS) values for CNS permeability were −2.434 and −3.136 for compounds **8** and **11**, respectively. Hepatic and renal clearance were assessed to evaluate overall drug clearance, calculated through the total clearance and elimination rate. Excretion rates are expressed in log (mL/min/kg). The anticipated ADME analysis results are summarized in Table 2.

### 2.5. In Silico Investigation of Physicochemical Properties for Oral Bioavailability and Drug Likeness for Compounds ***8*** and ***11***

**Figure 9:**
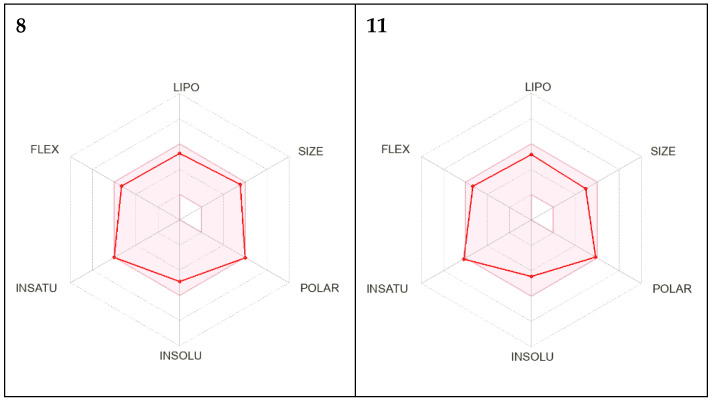
Oral bioavailability prediction for compounds **8** and **11**, where the red nets * represent the physicochemical space for oral bioavailability. * LIPO (Lipophilicity): −0.7 < XLOGP3 < +5.0; SIZE: 150 g/mol < MV < 500 g/mol; POLAR (Polarity): 20 Å^2^ < TPSA < 130 Å^2^; INSOLU (Insolubility): −6 < Log S (ESOL) < 0; INSATU (Instauration): 0.25 < Fraction Csp3 < 1; FLEX (Flexibility): 0 < Num. rotatable bonds < 9.

An in silico assessment of the tested compounds **8** and **11** was performed using Swiss ADME web. In terms of oral bioavailability (Figure 9), the screened compounds demonstrated a solubility (log S) score of around −4.0. The number of rotatable bonds was eight for both compounds compared to the standard range (one to nine). In terms of polarity, the topological polar surface areas (TPSAs) for the tested thiazolidine-2,4-dione **8** and its analogue structure **11** were 130.42 Å^2^ and 125.49 Å^2^, respectively, while the calculated standard range is 20–130 Å^2^ [29]. In reference to Lipinski’s rule of five, both compounds have no violations of the rule of Lipinski. In addition, no violations were recorded against the Ghose, Veber, Egan, and Muegge rules, except for the molar refractivity in compound **8**. The results are given in Table 3.

## 3. Discussion

The current study elucidated the potential capabilities of the two investigated thiazolidinedione and neocryptolepine analogues, i.e., thiazolidinedione–acridine (TZD-AC) and neocryptolepine–thiazolidinedione (NCL-TZD), in regulating persistent hyperglycemia and lipid deposition. Both characteristics are common manifestations related to the development of type 2 diabetes mellitus (T2DM). Zebrafish adults showed clear responses to persistent hyperglycemia induction, and to its treatment as well.

The upregulation of *nfκβ* in the positive control group aligns with the established role of hyperglycemia in significantly activating the expression of this key pro-inflammatory cytokine in various cell types [30,31]. This elevation indicates impaired β-cell function and identity without causing outright cell destruction [32], which is consistent with the reduction in insulin concentration observed in this positive control group. NF-κβ activation also leads to the production of other pro-inflammatory cytokines, such as TNF-α, IL-1β, and IL-6, as well as other inflammatory mediators like COX-2 and iNOS, all of which contribute to the development of a chronic inflammatory process [31,33]. Moreover, the subsequent production of pro-inflammatory and inflammatory cytokines secondary to NF-κβ upregulation is associated with elevated triglyceride levels and dysfunction of lipid metabolism [30,31], which is also consistent with the identified hypertriglyceridemia in the positive control group. Hypertriglyceridemia, as observed in the positive control group in the current study, usually precedes the release of more free fatty acids. These free fatty acids disrupt the glucose uptake cascade regulated by its transporters (such as GLUT4) and insulin signaling (for example, through insulin receptor 1). Hence, hypertriglyceridemia further complicates glucose metabolism disturbances, exacerbating the chronic inflammatory status and insulin resistance, along with β-cell dysfunction [34,35].

Therefore, the ability of the NCL analogue tested in the current study to reduce inflammation can be attributed to its capability to reduce *nfκβ*, chronic inflammation, and insulin resistance. The NCL-TZD compound was synthesized and tested for the first time in this study, revealing its actions for the first time. However, its parent compound showed somewhat similar results. In this context, synthetic cryplolepine could block the DNA binding activity of activated NF-κβ, thereby suppressing the transcription of NF-κβ-regulated inflammatory genes [36]. Moreover, NCL at doses comparable to those applied in this study exhibited dose-dependent ameliorative effects on beta cells, hyperglycemia, elevated free cholesterol, and low-density lipoproteins, as well as increasing the levels of high-density lipoproteins [37]. Additionally, cryplolepine reduced hyperglycemia by altering the α-glucosidase enzyme structure and function through direct binding [38,39]. Thus, cryptolepine administration improved glucose tolerance and reduced postprandial hyperglycemia, supporting its potential as an antidiabetic agent [38,39]. Hence, the enhanced insulin levels and reduced glucose and triglyceride levels in the NCL-TZD-treated groups in this study can be attributed to the *nfκβ* and α-glucosidase inhibitory effects, as well as the β islet ameliorative effects of this analogue.

Thiazolidinediones, however, perform their function in inhibiting *nfκβ* by targeting its upstream regulator, IKK-β, rather than precluding NF-κβ DNA binding, which is known for cryptolepine [40]. Moreover, TZDs and acridine-based compounds have shown strong inhibitory effects on the α-glucosidase enzyme. This inhibitory action works by binding to the enzyme and blocking its activity, thereby reducing glucose absorption and postprandial blood sugar spikes [41,42]. Some TZD hybrids also inhibit α-amylase [43,44]. As a result, inhibiting these two enzymes, especially α-glucosidase, lowers blood glucose levels [45]. Therefore, the reduction in glucose and triglyceride levels induced by different doses of TZD-AC could result from the potent inhibitory action of this hybrid compound on either or both of α-glucosidase and α-amylase.

Moreover, the current study assessed the expression of *acox1* and *accα* in a hyperglycemic zebrafish model in response to NCL and TZD hybrids. To our knowledge, no previous studies have assessed the regulation of these lipid-metabolizing enzymes in response to these treatments. Therefore, the direct role of NCL and TZD analogues assessed in its regulation is still in need of more studies. However, the reduction in *acox1* in the positive control group and its elevation in the TZD and NCL-treated groups in the current study align with the known roles of this enzyme in preventing triglyceride accumulation. Since Acox1 is involved in the β-oxidation of hydrolyzed triglycerides, its expression varies inversely with triglyceride levels [46]. Specific blockers of Acox1, such as microRNAs like miR222, have been correlated with a significant elevation of triglyceride levels [12]. Hence, the *acox1* upregulation and triglyceride reduction in the hybrid-treated group may point to the ameliorative role of these drugs in the active reduction in hypertriglyceridemia.

The antidiabetic properties of thiazolidinediones are primarily due to their function as agonists of the peroxisome proliferator-activated receptor gamma (PPARγ), a member of the nuclear receptor family. However, newer derivatives are being developed to act as dual agonists of both PPARα and PPARγ, offering potential benefits in treating conditions such as obesity and diabetic cardiomyopathy. This dual action influences downstream targets like Acox1 [47]. Therefore, the observed upregulation of *acox1*, along with reductions in total triglycerides and NF-κB transcription levels, provide an insight into how these compounds may modulate various inflammatory pathways. PPARα mainly regulates genes involved in lipid metabolism. It activates the expression of several key genes, including that for lipoprotein lipase, which facilitates the release of fatty acids from lipoprotein particles; CD36, also known as fatty acid translocase; and fatty acid-binding proteins, which assist in the uptake and transport of fatty acids across cell membranes—findings supported by Dixon et al. [48] and Tahri-Joutey et al. [49]. Another important gene target of PPARα is acyl-CoA synthetase, an enzyme responsible for converting fatty acids into acyl-CoA esters. This role has been highlighted in studies by Kersten and Stienstra (2017) [50] and Duszka et al. (2020) [51]. The activation of the peroxisomal fatty acyl-CoA transporter is mostly dependent on PPAR-α regarding genes encoding peroxisomal-oxidation enzymes [52]; nevertheless, the regulation of ThB, L-PBE, and ACOX1 is totally dependent on PPAR-α [48,49,51,53].

Accordingly, PPAR-α and ACOX1 were selected for molecular docking as targets for the synthesized dioxothiazolidine derivatives **8** and **11** to explore their potential antihyperlipidemic mechanism of action. A redocking step was performed to allocate the ligand at the binding site with its amino acid residues and to validate the docking process prior to testing the synthesized compounds, which revealed complete superimposed structures for both the co-crystallized ligand and the redocked one, with us recording an RMSD value not exceeding 2 Å. Docking of compounds **8** and **11** was then performed, where their binding affinities and RMSD values were promising and fitted totally inside the ligand binding cavity with hydrogen bonding and hydrophobic interactions as well as sharing amino acid residues with the corresponding co-crystalized ligand. Docking at PPAR-α demonstrated the interaction of compound **8** with two hydrogen bonds; one H-bond is between the amino group attached to the indoloquinoline structure and SER280 in the ligand binding pocket, and the other is between the amino acid of the thiazolidinone ring and MET355 in addition to three hydrophobic interactions. Meanwhile, compound **11** demonstrated two H-bonds between the thiazolidine ring attached to the substituted acridine structure and CYS 276 and MET 330. Docking at the ACOX1 binding pocket revealed the interaction of compound **8** with two hydrogen bonds, with GLN 138 and THR 139, in addition to hydrophobic interactions between the amino acid residues and the tetracyclic coplanar structure. Compound **11**, however, demonstrated a hydrogen bond between the dioxothiazole ring and GLN 138 amino acid in addition to hydrophobic interactions with the indoloquinoline skeleton. In addition, the inhibition constant (Ki) of the prepared hybrids **8** and **11** was calculated, and compound **8** showed a lower Ki than its analogue compound **11** against the two target proteins, which suggests that compound **8** is more potent than **11**.

Furthermore, analysis of absorption, distribution, metabolism, and excretion (ADME) is crucial for streamlining clinical trials, particularly during the early phases of drug development [25,26,27,28]. Key absorption parameters in drug discovery include intestinal absorption, skin sensitization, and oral bioavailability. An intestinal absorption score above 30% indicates optimal absorbance. Both compounds under study demonstrated intestinal absorption rates exceeding 30%, achieving impressive absorbance of 92% for compound **8** and 83% for compound **11.**

A compound is considered to have low skin permeability if its log *Kp* exceeds −2.5, and the compounds tested showed favorable skin permeability, with scores around −2.7 cm/h. Compounds are classified as having high permeability for human colon adenocarcinoma (Caco-2) when their Caco-2 value is greater than 0.9, indicating their ability to cross a monolayer of Caco-2 cells, which resemble small intestine epithelial cells. The compounds tested displayed a low Caco-2 permeability score of 0.6 for compound **8** and a slightly higher score of 1.0 for compound **11**, suggesting that compound **8** may have poorer absorption in the small intestine compared to compound **11**.

To analyze the distribution of the compounds in silico, parameters such as the volume of distribution (VDss), blood–brain barrier (BBB) permeability, and central nervous system (CNS) permeability were evaluated. The VDss values indicated distribution volumes of 0.165 and 0.35 log L/kg for compounds **8** and **11**, respectively. A log BB value below −1 indicates low BBB permeability; compound **8** had a log BB score of −0.987, indicating better BBB permeability than compound **11**, which had a score of −1.102. The log permeability surface (PS) values for CNS permeability were −2.434 for compound **8** and −3.136 for compound **11**. Since low CNS permeability is indicated by a log PS below −3, compound **8** shows potential for CNS permeability, whereas compound **11** is considered CNS impermeable.

Toxicity is a critical factor in drug design, which significantly influences the selection of viable candidates. In terms of AMES toxicity, a positive result indicates mutagenic and carcinogenic properties. Compound **11** exhibited mutagenic traits, suggesting potential cytotoxicity, while compound **8** showed no AMES toxicity; neither compound exhibited predicted hepatotoxicity or skin allergic reactions. hERG inhibition (I and II) is vital for toxicity assessment, including cardiotoxicity, and both compounds inhibited hERG I. Toxicity against T. pyriformis protozoa yielded IGC_50_ values of 0.444 and 0.387 µg/L for compounds **8** and **11**, respectively.

An in silico assessment of the physicochemical properties of the tested compounds **8** and **11** was performed. In terms of oral bioavailability, the screened compounds demonstrated optimum solubility (log S) (i.e., no higher than six). The number of rotatable bonds was eight, which lies within the required range (one to nine). In terms of polarity, the topological polar surface areas (TPSAs) for the tested thiazolidine-2,4-dione **8** and its analogue structure **11** were 130.42 Å^2^ and 125.49 Å^2^, respectively, which suggest an excellent oral bioavailability for both tested thiazolidine-2,4-diones as both compounds are within the calculated standard range [29]. Regarding drug likeness, both tested thiazolidinedione derivatives were promising. In reference to Lipinski’s rule of five, both compounds are free of violations of the rule of Lipinski as their hydrogen bond acceptors do not exceed ten, and the number of hydrogen bond donors does not exceed five. In addition, no violations were recorded of the Ghose, Veber, Egan, and Muegge rules, except for the molar refractivity in compound **8**, which slightly exceeded the required range (130). Lipophilicity should be ≤5, and both tested compounds showed an optimum lipophilicity in all terms, which indicated their high tolerability with cell membranes.

It is worth adding that, although zebrafish offer significant advantages for biomedical research, including rapid development, genetic tractability, and suitability for high-throughput screening, there are notable limitations when using them as substitutes for mammalian models. In this context, metabolic procedures such as hormone testing and insulin resistance tests in zebrafish are substantially more challenging, because of the limits of recurring blood samples [54]. The small size of the fish adds difficulty to the collection of adequate amounts of tissue and some body fluids as blood samples [54,55]. Also, it precludes drug administration methods, e.g., intraperitoneal and intravenous injection. Furthermore, the number of individuals per experimental group should be increased to include several metabolites/genes/measurements, as a single tissue cannot easily be divided to be used for different assays. However, zebrafish share a high degree of genetic similarity with humans, including the conservation of key metabolic pathways involved in glucose homeostasis, making them best suited to studying diabetes mellitus [56].

## 4. Materials and Methods

### 4.1. Characterization Techniques and Chemicals

Varian and Bruker Avance were used for all ^1^H- and ^13^C NMR experiments, which were carried out in deuterated dimethyl sulfoxide-d6 (DMSO-d6) with 400 MHz, at the main chemical warfare laboratories, Egypt. Chemical shifts are reported in parts per million (ppm) relative to the respective tetra methyl silane (TMS) as the internal standard. Fourier transform infrared spectroscopy (FTIR) was carried out at the Applied Nucleic Acid Research Center, Faculty of Science, Zagazig University using a Bruker Alpha II ATR mode spectrometer, Bremen, Germany, in the wavenumber range of 4000–400 cm^−1^. The sample was prepared using the KBr pellets technique. The mass spectrometry experiments were recorded on thermos scientific trace 1310 gas chromatograph at Fungi National Centre, Al-Azhar University, Egypt. The purity of all compounds was checked by thin-layer chromatography (TLC) on Kieselgel 60 F254 precoated plates (Merck KGaA, Darmstadt, Germany) in a suitable solvent system with UV visualization (λ = 254 nm). The commercially available starting materials, such as methyl 1H-indole-3-carboxylate (99.0%), 1,4-dimethylpiperazine (99.0%), *N*-chlorosuccinimide (98.0%), trichloroacetic acid (99.0%), *N*-methyl aniline (99.0%), 1,3 diaminopropane (99.0%), maleic anhydride (98.0%), thiourea (98.0%), phosphorus oxychloride (POCl_3_), dimethyl formamide (DMF), concentrated hydrochloric acid (HCl), and triethyl amine (TEA), were purchased from Sigma Aldrich, Cairo, Egypt and used as received without further purification. Solvents such as dichloromethane, diethyl ether, diphenyl ether, and 1,4-dioxane were purchased from Al Gomhoria Company, Egypt with a high degree of purity and used with no additional purification required. The starting key intermediates, 2,4-dioxothiazolidine-5-acetic acid **3**, 2,4-dioxothiazolidine-5-acetyl chloride **4** [57], N-(3-Aminopropyl)-5-methyl-5H-indolo[2,3-b]quinolin11-amine **7** [58,59], and N1-(acridin-9-yl)propane-1,3-diamine **10** [60], were synthesized according to the literature method.

### 4.2. Synthesis of Neocryptolepine and Acridine Analogues

*General procedure for the synthesis of the intermediate 11-aminopropylamino neocryptolepine and 9-aminopropylamino acridine derivatives* **7** *and* **10:**

11-Chloroneocryptolepine **5** or 9-chloroacridine **9** (1 mmol) and 5-fold molar excess of 1,3 di-amino propane **6** (5 mol) were heated together at 80–120 °C for 2 h until the complete consumption of the starting materials occurred, as monitored by TLC. The reaction was left to cool then poured onto ice/water, and the resulting solid was filtered off and dried to give compound **7** or **10**.

*Synthesis of N-(3-Aminopropyl)-5-methyl-5H-indolo[2,3-b] quinoline 11-amine* **7:**

Yield: 94%, yellowish solids; mp: 69–72 °C; (literature m.p: 69–71 °C. IR (KBr) vmax 3433, 2926, 2868, 2357, 2340, 1622, 1559, 1484, 1440, 1418, 1286, 1246, 1058, 752, 669 cm^−1^; ^1^H NMR (CDCl_3_, 600 MHz): d = 1.80 (2H, quint, *J* = 6.0 Hz), 3.01 (2 H, t, *J* = 6.0 H), 4.01 (2H, t, J = 6.0 Hz), 4.22 (3H, s), 7.17 (1H, t, *J* = 7.2 Hz), 7.18 (1H, brs), 7.31 (1H, t, *J* = 7.2 Hz), 7.41 (1H, t, *J* = 7.2 Hz), 7.61 (1H, d, *J* = 9.0 Hz), 7.67 (1H, td, *J* = 7.2, 1.2 Hz), 7.76 (1H, d, *J* = 7.8 Hz), 7.95 (1H, d, *J* = 7.8 Hz), 8.13 (1H, d, *J* = 8.4 Hz); ^13^C NMR (CDCl_3_,150.8 MHz): d = 32.64, 32.66, 41.26, 49.25, 105.61, 114.40, 115.86, 116.94, 118.49, 120.35, 121.37, 124.06, 124.12, 125.16, 130.08, 137.79, 148.52, 151.94, 156.55; HRMS (ESI) calcd for C_19_H_21_N_4_ [M^+^ H]^+^ exact mass: 305.1761, found 305.1770.

*Synthesis of N1-(acridin-9-yl) propane-1,3-diamine* **10:**

Yield: 97%, yellowish solid, m.p: 89–92 °C, (literature m.p: 88–90 °C). IR (KBr) cm^−1^ υ: 3416 (NH, NH_2_ overlap), 1646 2922 (C–H), 1646 (C=C, Ar), 1608 (C=N). ^1^H-NMR (CDCl_3_, 500 MHz), δ ppm: 11.62 (s, 1H, NH), 11.45 (s, 2H, NH_2_), 8.00–7.42 (m,8H, CH_Ar_), 3.64 (br.s,2H,CH_2_), 2.42 (m, 2H, CH2), 1.61 (m, 2H, CH_2_). ^13^C-NMR (DMSO-*d6*,100 MHz) δ: 161.53, 142.30, 129.75, 127.82, 126.38, 120.81, 114.84, 41.32, 39.42, 31.54. EIMS, *m*/*z* (C_16_H_17_N_3_) calcd, 251.33 [M] +; found 251.20.

*General procedure for synthesis of new hybrids* **8** and **11:**

A mixture of an equivalent 2,4-dioxothiazolidine-5-acetyl chloride **4** (4 mmol) and N-(3-Aminopropyl)-5-methyl-5H-indolo[2,3-b] quinoline 11-amine **7** or N1-(acridin-9-yl) propane-1,3-diamine **10** (4 mmol) was dissolved in dry DMF in the presence of a 3-fold excess of triethylamine, and the reaction mixture was refluxed for 6–8 h with constant stirring and monitored by TLC till completed. The mixture was poured onto crushed ice, and then the resulting precipitates were collected by suction filtration to obtain the crude products and further purified by recrystallized from ethanol.

*2-(2,4-Dioxothiazolidin-5-yl)-N-(3-((5-methyl-5H-indolo[2,3-b]quinolin-11-yl)amino) propyl) acetamide* **8**:

Dark brown, yield 63%, m. p 219–220 °C; IR (KBr, υ_max_, cm^−1^): 3360, 3324 (NH), 3125 (CH_Str.Ar-H._); 2967 (CH_Str.Aliph-CH._), 1772 (C=O); 1156 (C=S); 668 (C-S). ^1^H NMR δ ppm (DMSO-d6): 1.86 (m, 2H, -CH_2_ *J* = 6.0 Hz), 3.15 (d, 2H, -CH_2_ *J* = 6.0Hz), 3.39 (t, 2H, -CH_2_ *J* = 6.0 Hz), 3.4 (t, 2H, -CH_2_ *J* = 6.7 Hz), 3.42 (s, 3H, -CH_3_), 4.68 (t, 1H, -CH *J* = 6.5 Hz), 5.38 (br. s, 1H, NH), 7.70 (br. s, 1H, NH), 6.72–7.44 (m, 8H, CH_Ar_), 12.11 (br.s, 1H, NH); ^13^C-NMR (DMSO-*d_6_*,) δ: 29.9, 33.7, 35.1, 37.8, 41.2, 50,2, 97.2, 105.4, 107.5, 113.4, 124.8, 126.5, 127.1, 127.8, 128.3, 129.2, 140.4, 143.8, 146.7, 152.6, 167.1, 173.3, 175.2, EI-MS, *m*/*z*: (C_24_H_23_N_5_O_3_S) 461.5 [M]^+^.

*N-(Acridin-9-yl)-2-(2,4-dioxothiazolidin-5-yl)acetamide* **11:**

Light brown solid, yield 69%; m.p: >300 °C; IR(KBr) (υ,cm^−1^): 3565(NH), 3027 (=CH, Ar), 2941 (CH), 1743 (C=O), 1516 (C=C,_Ar_), ^1^H-NMR (DMSO-d_6_) δ: 1.85 (m, 2H, -CH_2_ *J* = 6.0 Hz), 2.9 (d, 2H, -CH_2_ *J* = 6.0 Hz), 3.35 (t, 2H, CH_2_ *J* = 6.7 Hz), 3.42 (t, 2H, CH_2_ *J* = 6.0 Hz), 4.68 (t, 1H, CH *J* = 6.6 Hz), 6.08 (br. s, 1H, NH), 7.80 (br. s, 1H, NH), 7.47–7.78 (m, 6H,CH_Ar_), 8.25 (d, 2H, 2 CH), 12.11 (br.s, 1H,NH); ^13^C-NMR (DMSO-*d_6_*), δ: 28.8, 33.7, 37.7, 41.3, 50,2, 114.8, 114.9, 120.7, 120.8, 126.2, 126.3, 127.8, 127.8, 129.7, 129.8, 142.3, 142.3, 161.5, 167.1, 173.3, 175.2, EI-MS, *m*/*z*: (C_21_H_22_N_4_O_3_S) 410 [M]^+^.

### 4.3. In Vivo Testing of Target Compounds

#### 4.3.1. Laboratory Acclimation of Experimental Fish

Adult mixed-sex *Danio rerio* zebrafish were purchased from commercial pet stores and kept for two weeks prior to the start of the experiment at standard water temperatures (22 ± 2 °C) and light:dark cycles (12 h:12 h). The fish were fed once a day with a diet for tropical fish aquaria, encompassing 23% crude protein, 3% crude fat, 1.5% crude fiber, 8.0% moisture, and 0.7% phosphorus. The feeding ratio was 2% of the total body mass daily. They were kept in 14 L plastic aquariums with mechanical and biological filters. Every day, 50% of the water was replaced after the tanks were cleaned to remove waste. Every day, the tanks were monitored for the possibility of mortality.

#### 4.3.2. In Vivo Experimental Procedures

All experimental protocol procedures were carried out in accordance with the instructions authorized by the Institutional Animal Care and Use Committee (IACUC) of the Department of Zoology at Menoufia University in Egypt’s Faculty of Science (approval number MUFSFGE225, DATE 2025-02-04). Feeding continued until the end of the experiment and ceased only a day before a transfer or sampling event. A group of zebrafish (n = 50, weight: 1.8 ± 0.4 g, length: 1.5 ± 0.5 cm) were placed in a 20 L aquarium containing 100 mM of glucose (D-glucose “dextrose monohydrate”, MF: C_6_H_12_O_6_·H_2_O, Cat. No. G002, piochem, Egypt) solution in water. Another group (n = 10) was maintained until the end of the experiment in a 4 L aquarium without glucose, to serve as negative controls. Both groups were maintained in their aquaria for 12 days. Later on, i.e., on day 13, all fish were transferred into new 4 L aquaria (n = 10 zebrafish/aquarium) containing glucose-free, dechlorinated waters, and maintained there for a further 1 day, with adequate group-specific labeling for each aquarium. Then, the groups that came from the initial glucose immersion tank were fed orally with either the fish feed alone or the fish feed mixed with the drug. In this sense, five groups were characterized: the first received a normal diet without any drug, serving as a possible hyperglycemia-positive control. The second and the third received 20 and 80 mg/kg fish weight of thiazolidinedione–acridine (TZD-AC), respectively. The fourth and the fifth groups received 20 and 80 mg/kg fish weight of neocryptolepine–thiazolidinedione (NCL-TZD). The doses were selected as the averages among different studies that confirmed the anti-inflammatory and antidiabetic effects of cryptolepine [61,62,63] and thiazolidinidione analogues [37,64,65].

Feeding proceeded for 4 days. Then, fish were euthanized by placing in ice-cold water (2–4 °C). Each fish was dissected, and the entire livers were separately collected and preserved using the same method mentioned in Galal-Khalaf et al. 2022 [48]. liver tissues of 5 fishes were removed and placed in 10 (*w*/*v*) of RNAlater (Invitrogen™ AM7021, Waltham, MA, USA) at 4 °C overnight, in order to be transferred to −20 °C the next day, where they were kept until subsequent gene expression. Livers of the remaining 5 fishes in each experimental group were placed in sterile 1.5 mL tubes containing 500 µL of ice-cold phosphate buffer (KH_2_PO_4_, pH = 7.2, 10 mM). These tubes were separately and immediately homogenized. The homogenates were then centrifuged at 10,000× *g* for 10 min at 4 °C. The supernatants were transferred to new sterile 1.5 mL tubes. These new tubes were stored at −80 °C until we measured glucose, triglycerides, and insulin concentrations. The number of biological replicates in each group was selected based on Galal-Khallaf et al.’s [66] work, which elucidated the adequacy of these fish numbers to show significant changes in pro-inflammatory cytokine gene expression and metabolite levels.

#### 4.3.3. Assessment of Energetic Metabolites Levels

Measurement of glucose levels was carried out using a commercial kit (Spinreact, cat. No. 1001191). Also, triglycerides were measured using a commercial kit from Spinreact (Cat. no. 41032). In both cases, KH_2_PO_4_ (10 mM, pH 7) was used as a blank. For each treatment, homogenates of control and hyperglycemia-induced zebrafish were administered in triplicate in a 96-well microplate. The standard error of measurement means between the three duplicates of the same sample was used to compute intra-specific errors. The spectrophotometric plate reader (TECAN, infinite F50, Mannedorf, Switzerland) was used to read the plates at 500 nm following the addition of the reagents to the sample, negative control, blank, and standard curve wells.

#### 4.3.4. Assessment of Insulin Levels

Insulin concentrations were measured using an Insulin ELISA kit (Cat. No.: IN374S, Calbiotech Inc., El Cajon, CA, USA). In brief, this kit is based on the solid-phase sandwich ELISA method. The samples and conjugate reagent (anti-insulin biotin and horseradish peroxidase (HRP) were added to wells coated with streptavidin. Insulin from the samples to be tested binds to the matched pair, forming a sandwich complex that is immediately immobilized on the plate due to biotin–streptavidin interactions. Through a washing step, unbound protein and HRP conjugate were washed off. The intensity of color produced from the addition of the substrate is proportional to the concentration of insulin in the samples. The measurement followed the procedures mentioned in the kit protocol and in Olsen et al. (2012) [67] for zebrafish, using liver homogenates instead of serum. The absorbance was read at 450 nm. The values were converted according to Knopp et al. [68] as 1 μIU/mL = 6.00 pmol/L.

#### 4.3.5. RNA Extraction and QPCR

After removal from RNA*later*™, the total RNA was extracted using a GeneJET RNA Purification Kit (Thermo Scientific, Waltham, MA, USA, Cat. No. K0731) without modifications to the tissue extraction protocol. RNA quantities and purities were quantified using spectrophotometric measurements at 260 nm and a 260/280 dual wavelength, respectively. Moreover, RNA quality was checked in ethidium bromide-stained RNase-free 1% agarose gel electrophoresis. With the best quality, purity (1.8–2), and quantity of RNA, 500 ng of total RNA was subjected to reverse transcription, using an ABT-H minus cDNA synthesis kit (Applied biotechnology, Ismailia, Egypt) following the protocol steps. Then, a quantitative polymerase chain reaction (QPCR) was carried out using 2X ABT SYBR Mix with low ROX (Applied biotechnology, Egypt), an Applied Biosystems 7300 Real-Time PCR System, and UltraFlux^®^flat 0.2 mL flat cap PCR strips (Cat. No. 3135-00, SSIbio, Lodi, CA, USA). Genes for enzymes involved in de novo lipogenesis (fatty acid synthase (fasn) and acetyl-CoA carboxylase alpha (*accα*)), adipogenesis (diacylglycerol O-acyltransferase 2 (dgat2)), and lipid catabolism through β-oxidation (acetyl-CoA oxidase 1 (*acox1*)) were the targeted transcripts for QPCR amplification in the current study. As an internal reference, beta actin (*actb*) was measured in each sample, based on its very low C_t_ differences among different samples. Sequences of all QPCR primers applied in the current study, besides the expected amplicon sizes and applied QPCR program, are mentioned in Table 4. Finally, a dissociation curve was appended to all PCRs to ensure the existence of a single amplicon for each transcript. To calculate the expression of target transcripts, the 2^−ΔΔCt^ method was applied.

### 4.4. Applied Data and Programs for Molecular Docking

The target protein was downloaded from a protein data bank, complexed with its co-crystalized ligand, with pdb ID: 2ZNN and 7Q86 for PPAR-α for *acox* 1, respectively. Preparation of both the ligand and protein was performed using UCSF-chimera as reported before [69,70]. An auto grid was used to generate a grid box, which was resized over the target protein. In the chimera, AutoDock vina was started for docking. The results were saved as pdbqt and then visualized using Biovia Discovery Studio v21.1.0.20298. The inhibition constant (ki) was calculated as the ΔG value, the following formula: Ki = exp(ΔG/(R*T)) R is the ideal gas constant 1.987 cal/(mol·K), and T is the absolute temperature in Kelvin.

### 4.5. Data Analysis

Statistical analyses for among-group significance in glucose, insulin, and reference gene-normalized mRNA expression of target transcripts were performed using a one-way analysis of variance (ANOVA). Differences were considered significant at *p* < 0.01. The least significant difference (LSD) was applied as a post-hoc test for accurate differentiation of significant groups for each parameter assessed. All these statistical analyses were carried out using Statgraphics Centurion XVI software (StatPoint Technologies, Inc., Warrenton, VA, USA. www.statgraphics.com).

### 4.6. In Silico Physicochemical and Pharmacokinetics Analysis

In silico studies were performed using Swiss *ADME* and pre-*ADMET* software pkcsm (https://biosig.lab.uq.edu.au/pkcsm/, accessed on 15 June 2025).

## 5. Conclusions

This study investigated the synthesis and performed an antihyperglycemic/antihyperlipidemic evaluation of two novel nitrogen-containing heterocyclic scaffolds: neocryptolepine–thiazolidinedione (NC-TZD) **8** and acridine–thiazolidinedione (AC-TZD) **11**, using an obesity-induced zebrafish model. Both hybrids **8** and **11** effectively restored glucose, triglyceride, insulin, and *nfκβ* levels to normal. However, only the lower doses restored peroxisomal acyl-CoA oxidase (*acox1*) levels, while the higher doses were less effective. Moreover, molecular docking studies were performed on PAPRα and acox-1. Docking of compounds **8** and **11** revealed promising binding affinities and RMSD values, with a total fit inside the ligand-binding cavity with hydrogen bonding and hydrophobic interactions as well as sharing amino acid residues with the corresponding co-crystalized ligand. In silico ADMET analysis suggested excellent oral bioavailability and drug likeness for both compounds.

These findings indicate that both hybrids exhibit promising antihyperglycemic and antihypertriglyceridemic effects, particularly at lower doses, underscoring their potential as therapeutic agents for managing T2DM.

Future recommendations: The synthesized hybrids have been confirmed as useful leads for the development of new antidiabetic agents, but the full mechanistic pathways remain to be explored. Future studies are scheduled to cover these topics: a mechanistic validation using gene expression analysis for key regulators in glucose and lipid metabolism combined with molecular dynamics and an in vitro enzyme inhibition assay, in addition to using nanoparticle-based delivery systems to enhance bioavailability and selectivity.

## Figures and Tables

**Table 1:** Docking results for compounds **8** and **11** on two target proteins.

Compound No.	PDB ID	ΔG (kcal/mol)	Ki (µM)	RMSD (Å)	Residue of Interaction	Type of Interaction
**8**	2ZNN	−9.17	0.27	1.3	MET 355 SER 280 PHE 273 CYS 276 LYS 358 TYR 464	H-bond H-bond H-*pi* *pi*-H *pi*-H *pi*-H
	7Q86	−8.51	0.575	1.3	THR 139 TYP 176 PHE 420 GLN 138 PRO 177	H-bond *Pi-pi* stack *Pi-pi* stack H-bond *Pi*-H
**11**	2ZNN	−7.89	1.89	1.8	CYS 276 MET 330	H-bond H-bond
	7Q86	−7.15	5.60	1.9	GLN 138 TRP 176 TYR 232 PHE 420 PRO 416	H-bond *Pi-pi* stack *Pi-pi* stack *Pi-pi* stack *Pi*-alkyl

**Table 2:** Calculating the predicted ADMET for compounds **8** and **11**.

Property	Model Name (Unit)	Predicted Value	
		Compound 8	Compound 11
Absorption	Intestinal absorption (human) (% absorbed)	92.6	83.729
	Caco2 permeability (log Papp in 10^−6^ cm/s)	0.606	1.088
	Water solubility (log mol/L)	−4.615	−4.173
	Skin permeability (log Kp)	−2.768	−2.752
	P-glycoprotein substrate	Yes	Yes
	P-glycoprotein I inhibitor	Yes	Yes
	P-glycoprotein II inhibitor	Yes	Yes
Distribution	VDss (human) (log L/kg)	0.165	0.35
	Fraction unbound (human) (Fu)	0.056	0.134
	BBB permeability (log BB)	−0.987	−1.102
	CNS permeability (log PS)	−2.607	−2.649
Metabolism	CYP2D6 substrate	No	No
	CYP3A4 substrate	Yes	Yes
	CYP1A2 inhibitor	No	Yes
	CYP2C19 inhibitor	Yes	Yes
	CYP2C9 inhibitor	Yes	Yes
	CYP2D6 inhibitor	No	No
	CYP3A4 inhibitor	Yes	Yes
Excretion	Total clearance (log mL/min/kg)	0.416	0.285
	Renal OCT2 substrate	No	No
Toxicity	AMES toxicity	No	Yes
	Max. tolerated dose (human) (log mg/kg/day)	−0.473	0.322
	hERG I inhibitor	No	No
	hERG II inhibitor	Yes	Yes
	Oral rat acute toxicity (LD50) (mol/kg)	2.446	2.502
	Oral rat chronic toxicity (LOAEL) (log mg/kg_bw/day)	1.129	1.229
	Hepatotoxicity	Yes	Yes
	Skin sensitization	No	No
	*T. pyriformis* toxicity	0.444	0.387
	Minnow toxicity	4.187	2.79

**Table 3:** Physicochemical properties of compounds **8** and **11**.

	Compound 8	Compound 11
*Physicochemical Properties*		
Formula	C_24_H_23_N_5_O_3_S	C_21_H_20_N_4_O_3_S
Molecular weight	461.54 g/mol	408.47 g/mol
Num. heavy atoms	33	29
Num. arom. heavy atoms	17	14
Fraction Csp3	0.25	0.24
Num. rotatable bonds	8	8
Num. H-bond acceptors	4	4
Num. H-bond donors	3	3
Molar refractivity	134.95	118.19
TPSA	130.42 Å^2^	125.49 Å^2^
*Lipophilicity*		
Log *P*_o/w_ (iLOGP)	2.30	1.82
Log *P*_o/w_ (XLOGP3)	3.73	3.55
*Hydrophilicity*		
Log *S* (ESOL)	−4.90	−4.44
Solubility	5.75 × 10^−3^ mg/mL; 1.25 × 10^−5^ mol/L	1.49 × 10^−2^ mg/mL; 3.65 × 10^−5^ mol/L
Class	Moderately soluble	Moderately soluble
*Drug likeness*		
Lipinski	Yes; 0 violation	Yes; 0 violation
Ghose	1 violation: MR > 130	Yes
Veber	Yes	Yes
Egan	Yes	Yes
Muegge	Yes	Yes
Bioavailability score	0.55	0.55

**Table 4:** Sequences of all QPCR primers, besides the expected amplicon sizes applied in the current study.

Locus ID	Gene	Amplicon Size (bp)	Forward Primer (5′-3′)	Reverse Primer (5′-3′)
NM_001003414	*nf-κB*	202	GGTCGGACAGAGATCACGGATT	TGCTGTTCTTCACGTCCTCT
NM_001271308.1	*accα*	194	AGAGAGGGCAGGTTTTACCA	GCCATCATACGAGAGCAACA
BC097101.1	*acox1*	162	GCACGGATGTGTGTACCGTGC	GCGTCCAGAGCCCCTTGACCT
NM_181601.3	*actb*	197	ACTGTATTGTCTGGTGGTAC	TACTCCTGCTTGCTAATCC

## Data Availability

All data are included in this manuscript.
